# Genome-wide association study of depression symptoms using online self-questionnaires in the Russian population cohort: preliminary results

**DOI:** 10.1192/j.eurpsy.2022.832

**Published:** 2022-09-01

**Authors:** A. Kibitov, A. Rakitko, E. Kasyanov, D. Yermakovich, A. Kibitov, G. Rukavishnikov, V. Ilinsky, V. Golimbet, A. Shmukler, N. Neznanov, G. Mazo

**Affiliations:** 1 Serbsky National Medical Research Center on Psychiatry and Addictions, Molecular Genetics Laboratory, Moscow, Russian Federation; 2 Genotek Ltd., N/a, Moscow, Russian Federation; 3 Bekhterev National Medical Center for Psychiatry and Neurology, Translational Psychiatry, Saint-Petersburg , Russian Federation; 4 Bekhterev National Medical Research Center for Psychiatry and Neurology, Translational Psychiatry Department, Saint-Petersburg , Russian Federation; 5 Mental Health Research Center, Clinical Genetics Laboratory, Moscow, Russian Federation; 6 Serbsky National Medical Research Center on Psychiatry and Addictions, Department Of Psychiatry, Moscow, Russian Federation; 7 Bekhterev National Medical Center for Psychiatry and Neurology, Geriatric Psychiatry, Saint-Petersburg , Russian Federation

**Keywords:** Depression, GWAS, phenotyping

## Abstract

**Introduction:**

Depression is a chronic, recurrent mental disorder with a moderate level of genetic impact. Modern GWAS of depression require extra-large sample sizes and new effective, clinically sensitive, objective and simple to fill online phenotyping tools for population studies are necessary today.

**Objectives:**

Aim: to test online phenotyping tools based on clinical and psychometric instruments to evaluate depressive symptoms in population cohort for using in GWAS

**Methods:**

Participants: 2610 Russian-speaking respondents- clients of Genotek Ltd., provider of genetic testing services in Russian Federation. The online survey included HADS-D (Hospital Anxiety and Depression Scale - depression subscale), original questionnaire adapted for self-report from major depression DSM-5 criteria, questions about sex and age. Three research phenotypes were defined: quantitative “HADS-D score” and two categorical “HADS-D depression” (cut-off 8 points) **-** 20.63 %, “DSM depression” 16.73 %. DNA samples obtained from saliva were genotyped on Illumina Infinium GSA v1.0/v2.0/v3.0 microarrays. GWAS analysis was performed independently for each of the research phenotypes.

**Results:**

None of the signals reached genome-wide significance (p value 10-8), but some signals with subthreshold significance were identified including four signals in the genes encoding proteins: “DSM_Depression”: rs2131596 in *GRIP1* (p=3.682e-06,β=0.6965), rs11158021 in *SAMD4A* (p=2.841e-06, β=0.6762), “HADS-D Depression”: rs2425793 in *CDH22* (p=4.408e-07, β=1.539), rs36006890 in *PDIA6* (p = 6.529e-07, β=1.549).

**Conclusions:**

Preliminary results of the first GWAS of depression symptoms in the Russian population are acceptable and confirm the accuracy of the research strategy using online phenotyping tools based on clinical and psychometric instruments and provide basis for further studies.

**Disclosure:**

The study was supported by Russian Science Foundation Grant # 20-15-00132

